# Neurological update: MOG antibody disease

**DOI:** 10.1007/s00415-018-9122-2

**Published:** 2018-12-19

**Authors:** Ray Wynford-Thomas, Anu Jacob, Valentina Tomassini

**Affiliations:** 1 Division of Psychological Medicine and Clinical Neurosciences, Cardiff University School of Medicine, University Hospital of Wales, Heath Park, Cardiff, CF14 4XN UK; 2 Helen Durham Centre for Neuroinflammation, University Hospital of Wales, Cardiff, UK; 3 Walton Centre NHS Foundation Trust, Liverpool, UK; 4 Cardiff University Brain Research Imaging Centre (CUBRIC), School of Psychology, Cardiff, UK

**Keywords:** Myelin oligodendrocyte glycoprotein (MOG) antibodies, Multiple sclerosis (MS), Neuromyelitis optica (NMO), Optic neuritis (ON), Cerebrospinal fluid (CSF), MRI

## Abstract

Myelin oligodendrocyte glycoprotein (MOG) antibody disease (MOG-AD) is now recognised as a nosological entity with specific clinical and paraclinical features to aid early diagnosis. Although no age group is exempt, median age of onset is within the fourth decade of life, with optic neuritis being the most frequent presenting phenotype. Disease course can be either monophasic or relapsing, with subsequent relapses most commonly involving the optic nerve. Residual disability develops in 50–80% of patients, with transverse myelitis at onset being the most significant predictor of long-term outcome. Recent advances in MOG antibody testing offer improved sensitivity and specificity. To avoid misdiagnosis, MOG antibody testing should be undertaken in selected cases presenting clinical and paraclinical features that are felt to be in keeping with MOG-AD, using a validated cell-based assay. MRI characteristics can help in differentiating MOG-AD from other neuroinflammatory disorders, including multiple sclerosis and neuromyelitis optica. Cerebrospinal fluid oligoclonal bands are uncommon. Randomised control trials are limited, but observational open-label experience suggests a role for high-dose steroids and plasma exchange in the treatment of acute attacks, and for immunosuppressive therapies, such as steroids, oral immunosuppressants and rituximab as maintenance treatment.

## Introduction

Myelin oligodendrocyte glycoprotein (MOG) is a glycoprotein located on the myelin surface and found exclusively in the central nervous system (CNS) [1, 2]. Although its exact role remains unclear, it is thought to act as a cellular adhesive molecule, to be involved as a regulator of oligodendrocyte microtubule stability and to mediate complement cascade [3].

MOG antibodies have been extensively studied over the last 30 years, with some early experimental studies hypothesising a pathogenic role in CNS inflammatory diseases [4, 5]. This hypothesis was later supported by their discovery in the sera and cerebrospinal fluid (CSF) of patients with multiple sclerosis (MS), using techniques such as enzyme-linked immunosorbent assay and Western blot [6–11]. However, the more recent development of highly sensitive and specific methods for MOG antibody detection using cell-based assays, along with new diagnostic classification of similar neuroinflammatory conditions, has made it possible to identify a subset of patients with antibodies to MOG who express a clinical phenotype distinct from MS or from neuromyelitis optica (NMO) [12–15]. Therefore, MOG antibody disease (MOG-AD) is now recognised as a distinct nosological entity with specific management and therapeutic requirements. In this article, we review the clinical features, investigations and challenges of managing patients with MOG-AD.

## Clinical features of MOG antibody disease

MOG-AD is an inflammatory demyelinating condition of the CNS characterised by a monophasic or relapsing course of neurological dysfunction, which does not meet the typical criteria for MS or other known neuroinflammatory conditions and occurs in the presence of serum MOG antibodies detected using specific cell-based assays [16].

Although in most cases demyelination associated with MOG antibodies occurs without any apparent inciting or predisposing event/illness, it has been associated with demyelinating *N*-methyl-d-aspartate receptor encephalitis, post-infectious demyelination following herpes simplex virus, Borrelia and Epstein–Barr virus infections and, more rarely, with typical relapsing MS [17–27]. Whether MOG antibodies play a pathogenic role in all these conditions, or if they represent a bystander effect or epiphenomenon, remains unclear [28].

MOG-AD can occur in all decades of life, with a slight predominance in women and with median age of onset in the early to mid-thirties [17, 18, 29–33]. The most common presenting feature is optic neuritis (ON), occurring in 54–61% of patients, followed by myelitis, acute disseminated encephalomyelitis (ADEM) or an ADEM-like presentation (e.g., brainstem attack) [18, 29, 31, 32].

A relapsing course has been reported in 44–83% of patients [18, 29, 32] and more commonly involves the optic nerve [30–32]. MOG-positive ON is frequently bilateral and associated with optic nerve head swelling [34, 35]. The impact of relapses on disability is variable: some studies report no difference between monophasic and relapsing disease courses, [29] whilst others report worsening disability associated with higher relapse frequency [32]. In case-based series, residual disability develops in 50–80% of patients, [18, 31, 32] with transverse myelitis at onset being the most significant predictor of long-term disability [32]. Several case-based series of seizures associated with MOG-AD have also been reported [36–38].

At presentation, MOG-positive patients are thought to be at lower risk of further relapses than aquaporin 4 (AQP4)-positive patients [29] and have better visual and motor outcomes [29, 32]. In comparison to children with AQP4-positive neuromyelitis optica spectrum disorder (NMOSD), those with MOG-AD tend to be younger, less likely to present with area postrema syndrome, but more likely to present with ADEM. In addition, they tend to have a longer time to relapse and lower disability at follow-up (2 years) [17]. Compared to AQP4-positive ON, MOG-positive patients have better visual field outcomes, [39] although recurrence of ON is significantly more frequent [40].

Table 1 summarises the clinical and paraclinical features of MOG-AD, as well as the characteristics that help in distinguishing MOG-AD from more common forms of neuroinflammation, i.e., MS and NMO.

**Table 1:** Clinical and paraclinical features of MOG-AD, NMO and MS

Characteristics	MOG-AD	NMO [41]	MS [41]
Age of onset	Early to mid-30s	Around 40	Around 30
Sex	Slight predominance in women	AQP4 −NMO: equal distribution AQP4 + NMO: more common in women	More common in women
Clinical phenotype	Commonly ON at onset (better visual field outcomes compared to AQP4-positive ON); other presentations include myelitis, ADEM and ADEM-like events	ON, usually severe with limited recovery; transverse myelitis; intractable nausea with hiccups or vomiting	ON, usually with good recovery; other neurological systems involved
Disease course	Monophasic or relapsing	Relapsing	Relapsing or progressive
Type of relapses	Commonly ON (more than in NMO)	ON; LETM	Any, with relapse phenotype predicted by previous relapse phenotypes [42]
MRI brain	Abnormal in 45–77%; fluffy T2 hyperintense lesions; few lesions (e.g., ≤ 3); bilateral lesions at onset (about 50% of cases); Dawson’s fingers and U- or S-shape lesions uncommon; thalamic and pontine lesions more common compared to NMO; more oedematous and extensive inflammatory lesions in the optic nerve, sparing chiasm and optic tracts; in children, cerebellar peduncle lesions	Abnormal in 60%; T2 hyperintense lesions around the 3rd and 4th ventricle and the aqueduct of Sylvius [43]; area postrema lesions	Always abnormal; presence of Dawson’s fingers, sub-cortical S-shaped or U-fibre lesions [44]
MRI spinal cord	Abnormal in about 50% of cases; lesions more commonly short; in children, LETM more common	LETM (≥ 3 vertebral segments) [45]	Lesions more commonly short [44]
CSF	Pleocytosis variable; OCBs uncommon	Commonly ≥ 50 WCC/mm^3^; glial fibrillary acidic protein at relapse; OCBs in 10–25%	Commonly < 50 WCC/mm^3^; OCBs in up to 95% of RRMS patients

*MOG-AD* myelin oligodendrocyte glycoprotein antibody disease, *NMO* neuromyelitis optica, *MS* multiple sclerosis, *ON* optic neuritis, *ADEM* acute disseminated encephalomyelitis, *LETM* longitudinally extensive transverse myelitis, *MRI* magnetic resonance imaging, *CSF* cerebrospinal fluid, *OCBs* oligoclonal bands, *WCC* white cell count, *RRMS* relapsing–remitting multiple sclerosis

## Paraclinical investigations for MOG antibody disease

### Magnetic resonance imaging (MRI)

Brain MRI scans are abnormal in approximately 45% of patients at onset [29, 31], with percentages increasing later in the course of the disease (i.e., up to 77% of patients) [18]. The majority have bilateral lesions at onset [29, 30] and around one-third have sub-tentorial lesions [18], predominantly in the brainstem [29]. Typically, lesions are few (three or less) and appear as “fluffy”, i.e., poorly demarcated hyperintensities on T2-weighted images. Dawson’s fingers, U- or S-shaped lesions and ovoid lesions adjacent to the body of lateral ventricles are found less commonly [29, 46]. When compared to MS-associated ON or AQP4-positive NMOSD-associated ON, the MRI appearance of the optic nerve in MOG-associated ON is more oedematous and shows extensive inflammatory lesions, usually sparing chiasm and optic tracts [35]. Thalamic and pontine lesions are more common in MOG-AD compared to AQP4-positive disease [29]. In children, bilateral thalamic lesions at onset are frequent and can be found in about 60% of patients [30]. Compared to AQP4-positive patients, cerebellar peduncle lesions are only found in MOG-positive children [17].

Just over half of MOG-AD patients have T2 hyperintense lesions in the spinal cord, with most lesions being short and predominantly occurring in the cervical or thoracic region [18]. In a study that explored MRI appearances in children, around 27% had abnormal initial spinal cord MRI scans, two-third of which showed a longitudinally extensive transverse myelitis (LETM) [30].

### Cerebrospinal fluid

CSF pleocytosis occurs in 44–85% of patients [18, 29, 31, 32] and is more common in children [30, 31]. Positive oligoclonal bands (OCBs) are unusual, occurring in only 6–17%, [18, 29–32] and CSF protein is raised in around a third of cases [31].

### Serum

It is currently recommended that MOG antibody immunoglobulin G (IgG) is detected in serum, using a cell-based assay (indirect fluorescence test or fluorescence-activated cell sorting) and employing full length human MOG as the target antigen [12, 13, 47]. As false positive MOG antibody results can occur, testing for MOG antibodies should be restricted to selected cases only, whereby the clinical and paraclinical features are felt to be in keeping with MOG-AD [16].

MOG antibody titres have been found to be higher in relapse than in remission [29]. A decreasing titre is usually found in a monophasic disease course and conversion to antibody negativity, which has been shown to occur within around 8–36 months from an acute event, has been associated with no further relapses [18, 32]. However, following a negative result, antibodies can become positive again, even after a few years [32, 47]. In children, persisting high-titre MOG antibodies (e.g., over 24 months) are associated with a risk of relapse [20].

There is no definite consensus regarding regular antibody monitoring. However, as antibodies can increase in relapse and can subsequently become negative, it could be argued that there is a role for regular monitoring at diagnosis, as well as throughout the course of MOG-AD, when reduced antibody levels may indicate disease remission. Some have suggested that a re-test interval of 6–12 months may be helpful [16]. Table 2 summarises when MOG antibody testing is indicated.

**Table 2:** When to test for MOG antibody

Test if	Clinical/paraclinical features are suggestive of MOG-AD (2018 International Recommendations) [16]
	Diagnosis of MS is made, interferon beta or natalizumab has been started, but efficacy is unexpectedly poor and clinical/paraclinical features are compatible with MOG-AD
Re-test if	MOG antibody positive, but clinical/paraclinical features not suggestive of MOG-AD (see “MOG antibody positivity and the diagnosis of MOG-AD: “red flags””)
	Clinical/paraclinical features continue to be suggestive of MOG-AD, but MOG antibody is negative
	Likelihood of further events is sought, following MOG-AD diagnosis

*MOG* myelin oligodendrocyte glycoprotein, *MOG-AD* myelin oligodendrocyte glycoprotein antibody disease

### Biopsy

In brain lesions of patients with positive MOG antibodies and the suspicion of MOG-AD, neuropathology seems compatible with pattern II histopathology of MS, i.e., with lesions presenting complement and IgG deposits at the sites of ongoing demyelination [22, 48].

## MOG antibody positivity and the diagnosis of MOG-AD: “red flags”

The 2018 International Recommendations have highlighted “red flags” that should prompt a physician to doubt the relevance of a positive result [16]. In these cases, re-testing is advised, preferably using a different cell-based assay, and expert-opinion should be sought. “Red flags” include progressive disease; sudden onset of symptoms or continuous worsening of symptoms over weeks; combined central and peripheral demyelination, as MOG is not expressed in the peripheral nervous system; a MRI lesion adjacent to the lateral ventricle that is ovoid/round or associated with an inferior temporal lobe lesion, or Dawson’s finger-type lesions; an active brain MRI over time with silent increase in lesion burden between relapses; bi- or tri-specific measles, rubella and zoster virus reaction in the CSF; serum MOG-IgG levels at or just barely above the assay-specific cut-off, especially if the clinical picture is atypical; positive MOG-IgM and/or MOG-IgA result with negative MOG-IgG; MOG-IgG positivity in the CSF, but not in the serum, given that MOG-IgG is typically produced extra-thecally; AQP4-IgG/MOG-IgG “double-positive” test results, which are very rare and should prompt re-testing for both antibodies [16]. In all these cases, caution should be exerted in diagnosing MOG-AD and clinical and paraclinical follow-up is advised.

## Pharmacological management of MOG antibody disease

Thus far, there are no controlled treatment trials in MOG-AD and observational, open-label experience is limited. Therefore, care must be taken when interpreting or extrapolating results from studies or reports. Due to these limitations, current treatment protocols for MOG-AD tend to follow those of AQP4− NMO.

### Acute treatment

Intravenous methylprednisolone (IVMP) for the treatment of MOG antibody positive ON and/or myelitis (dose ranging from 1 to 2 g once a day for 3–5 days) has been reported to be effective in some cases, with partial or no recovery in 50%. Even if treatment with IVMP is effective initially, further courses may not be [33].

Plasma exchange (regime varying from three to five cycles) is more commonly used as a second-line treatment following steroid resistance and can result in around 40% of cases of MOG antibody ON and/or myelitis having a complete or almost complete recovery [33].

### Disease-modifying treatment

Long-term treatments with prednisolone (dose > 10 mg/day for patients > 40 kg in weight and > 5 mg for patients ≤ 40 kg in weight [31]), intravenous immunoglobulins (induction course dose of 2 g/kg with subsequent monthly doses of 1 g/kg/infusion [31]), rituximab, mycophenolate mofetil, methotrexate or azathioprine have all been reported to reduce annualised relapse rate in MOG-AD [31, 33, 37]. However, treatment with rituximab can be associated with new attacks within the few weeks following the first infusion, possibly due to a temporary increase in B-cell activating factor and autoantibody levels, as also observed in some AQP4-positive NMO patients [33]. Although numbers are small, treatment with natalizumab has been shown to be ineffective in preventing relapses, whereas treatment with ofatumumab was able to reduce annualised relapse rate from 2.1 to 0.66 [33]. One study found that the rate of treatment failure was lower in patients on maintenance steroids (5%; median treatment duration of 10 months) compared to non-steroidal maintenance therapies (38%) [31].

The role of oral steroids as adjunctive treatment with immunosuppressants remains unclear. A study found that patients with MOG antibody ON and/or myelitis treated with azathioprine, but not adjunctively with oral steroids, experienced relapses more commonly than patients who underwent combination therapy [33]. Furthermore, relapses seem to occur more frequently when oral prednisolone doses are dropped below 10 mg/day or early after steroid cessation [31, 32].

Treatment with interferon beta has been shown to be ineffective and can increase disease activity [33, 37]. Similarly, glatiramer acetate has been shown to be ineffective when administered in children [37].

**Fig. 1:**
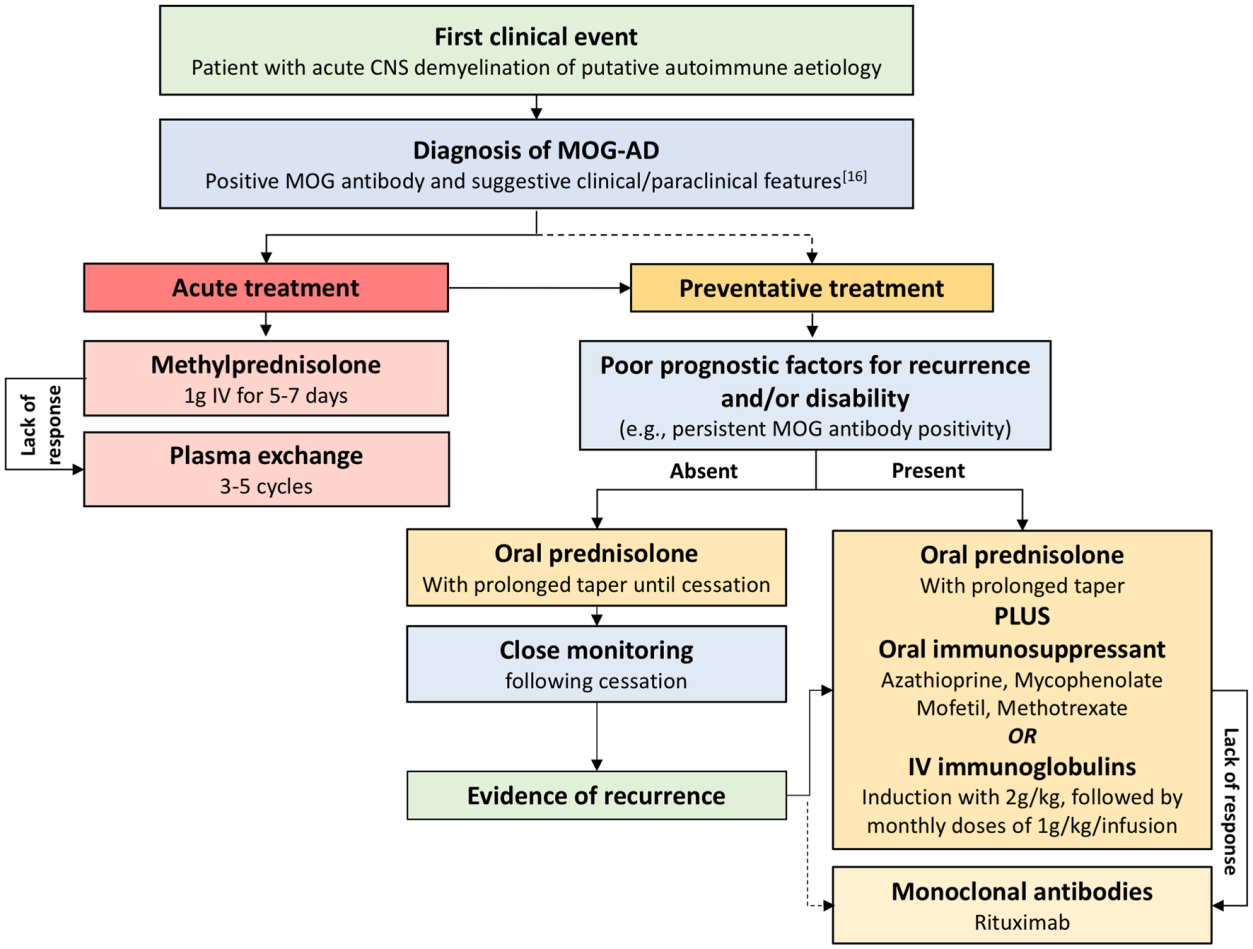
Management of MOG-AD. This proposed therapeutic management of MOG-AD is based on current evidence. Acute treatment with steroids and, if needed, subsequent plasma exchange are advised as soon as possible after an acute event. After diagnosis and, usually, acute treatment, initiation of disease-modifying therapy is advised. The choice of the disease-modifying agent should be guided by the presence or absence of poor prognostic factors for recurrence and/or disability. On this basis, treatment may require a prolonged taper with oral steroids (advised in the absence of poor prognostic factors) or the use of oral immunosuppressants or intravenous immunoglobulins along with oral steroids (advised as first choice in the presence of poor prognostic factors). If a lack of response to immunosuppressants is demonstrated or a disabling recurrence of the disease occurs after cessation of oral steroids, it is appropriate to consider monoclonal antibodies. While it is reasonable and common practice to treat relapsing patients with long-term immunosuppression, the duration of disease-modifying treatment remains uncertain. *CNS* central nervous system, *MOG-AD* myelin oligodendrocyte glycoprotein antibody disease, *MOG* myelin oligodendrocyte glycoprotein, *IV* intravenous

Figure 1 summarises the proposed management of MOG-AD, based on current evidence.

## Conclusions

The diagnosis of MOG-AD is crucial to plan appropriate management. Suggestive clinical and paraclinical features, in association with MOG antibody positivity, aid the identification of MOG-AD from other inflammatory demyelinating diseases, but, in many cases, a definitive diagnosis remains difficult. Therefore, the definition of MOG-AD-specific diagnostic criteria, as well as the identification of markers of disease status, remains a goal of research. Once the disease has been diagnosed, uncertainty remains over the best treatment approach and clinical trials for the pharmacological management of MOG-AD are still needed.
